# H-1 Parvovirus-Induced Oncolysis and Tumor Microenvironment Immune Modulation in a Novel Heterotypic Spheroid Model of Cutaneous T-Cell Lymphoma

**DOI:** 10.3390/cancers16152711

**Published:** 2024-07-30

**Authors:** Assia Angelova, Milena Barf, Alexandra Just, Barbara Leuchs, Jean Rommelaere, Guy Ungerechts

**Affiliations:** 1 Clinical Cooperation Unit Virotherapy, Infection, Inflammation and Cancer Program, German Cancer Research Center, 69120 Heidelberg, Germany; m.barf@dkfz-heidelberg.de (M.B.); a.just@dkfz-heidelberg.de (A.J.); j.rommelaere@dkfz-heidelberg.de (J.R.); guy.ungerechts@nct-heidelberg.de (G.U.); 2 Biopharmaceutical Production and Development Unit, German Cancer Research Center, 69120 Heidelberg, Germany; b.leuchs@dkfz-heidelberg.de; 3 Department of Medical Oncology, National Center for Tumor Diseases Heidelberg and Heidelberg University Hospital, 69120 Heidelberg, Germany

**Keywords:** cutaneous T-cell lymphoma (CTCL), virotherapy, oncolytic H-1 parvovirus, heterotypic CTCL spheroid

## Abstract

**Simple Summary:**

Cutaneous T-cell lymphoma (CTCL) is a rare type of T-lymphocyte malignancy strongly calling for novel therapies. Virotherapy by means of oncolytic viruses that are able to kill cancer cells, while sparing healthy cells, is a promising innovative form of anticancer immunotherapy. The aim of this study was to investigate the potential of an oncolytic parvovirus, H-1PV, to induce selective killing (oncolysis) of CTCL cells and suppress the growth of CTCL spheroids. We demonstrated that H-1PV treatment led to oncolysis in tumor, but not in control normal cells. Oncolysis ensued despite pro-survival protein overexpression and was associated with the release of danger-signaling molecules. In heterotypic CTCL spheroids, H-1PV induced spheroid growth suppression and, upon co-culturing with peripheral blood mononuclear cells, spheroid infiltration with immune cells. In summary, we gathered the first preclinical data showing that H-1PV holds significant potential to become a novel viroimmunotherapeutic agent against CTCL.

**Abstract:**

The rat protoparvovirus H-1 (H-1PV) is an oncolytic virus known for its anticancer properties in laboratory models of various human tumors, including non-Hodgkin lymphomas (NHL) of B-cell origin. However, H-1PV therapeutic potential against hematological malignancies of T-cell origin remains underexplored. The aim of the present study was to conduct a pilot preclinical investigation of H-1PV-mediated oncolytic effects in cutaneous T-cell lymphoma (CTCL), a type of NHL that is urgently calling for innovative therapies. We demonstrated H-1PV productive infection and induction of oncolysis in both classically grown CTCL suspension cultures and in a novel, in vivo-relevant, heterotypic spheroid model, but not in healthy donor controls, including peripheral blood mononuclear cells (PBMCs). H-1PV-mediated oncolysis of CTCL cells was not prevented by Bcl-2 overexpression and was accompanied by increased extracellular ATP release. In CTCL spheroid co-cultures with PBMCs, increased spheroid infiltration with immune cells was detected upon co-culture treatment with the virus. In conclusion, our preclinical data show that H-1PV may hold significant potential as an ingenious viroimmunotherapeutic drug candidate against CTCL.

## 1. Introduction

Cutaneous T-cell lymphoma (CTCL) is a rare type of primary cutaneous lymphoma that derives from mature, skin-resident, or skin-homing CD4+ T-lymphocytes. The most common CTCL subtypes, representing about 70% of all CTCL cases, are mycosis fungoides (MF) and Sézary syndrome (SS). Early-stage MF is an indolent disease characterized by the proliferation of malignant epidermotropic T-cells into the upper skin layers, leading to the formation of patches, plaques, and tumors. SS may arise de novo or progress from long-standing MF. In contrast to MF, SS is an aggressive type of CTCL, with characteristic systemic involvement with malignant CD4+ T-lymphocytes that are clonally related to those in the affected skin [1]. SS-associated generalized exfoliative dermatitis and fissure formation, with the related protein and electrolyte loss, hypothermia and ectropion, severely affect patients’ quality of life [2]. SS patient prognosis is strongly dependent on disease stage at the time of diagnosis, and several factors, such as large cell transformation, are known to be associated with poor outcome [3]. Current CTCL treatment options include topical steroids, Toll-like receptor agonists, phototherapy, radiation, and systemic chemotherapies in advanced-stage patients [4]. The treatment of advanced-stage MF/SS remains, however, largely palliative. Moreover, the multidisciplinary sequential approach required to manage advanced disease is associated with life quality-reducing side effects [4]. Novel therapeutic options are therefore urgently needed in order to improve the management of this malignancy.

Virotherapy by means of oncolytic viruses (OVs) that are able to induce selective killing—oncolysis—of tumor, but not of healthy cells, is an innovative anticancer approach recently gaining increasing consideration. After the groundbreaking U.S. Food and Drug Administration approval of talimogene laherparepvec (Imlygic^®^) [5], a plethora of other naturally occurring or manipulated OVs have been tested in preclinical and clinical settings against various types of human tumors [6]. At present, the capacity of OVs to selectively induce cancer cell death and stimulate antitumor immune responses has been documented mostly in patients with solid cancer types. In fact, oncolytic virotherapy remains poorly investigated in many human blood cancers, in particular those of T-cell origin. A scarce number of reports have been previously published that describe the induction by the oncolytic measles virus of CTCL cytolysis in vitro [7] and the mounting of an antitumor immune response in CTCL patients who received measles virus treatment [8]. Despite these encouraging results, to the best of our knowledge, further studies that explore the potential of oncolytic virotherapy in CTCL treatment are not available in the current literature. The therapeutic applications of OVs in CTCL comprise, therefore, a largely underexplored research niche.

The present study aims to undertake the first preclinical investigation of the potential of an oncolytic protoparvovirus, H-1 (H-1PV), to induce oncolysis and exert immunomodulation in CTCL. H-1PV, whose natural host are rats, is the smallest among all known OVs, with an overall diameter of the icosahedral capsid of about 18–26 nm and a genomic size of about 5 kb [9]. The virus was first detected in the middle of the last century in transplantable human tumors and was, as a matter of fact, initially suspected to be an oncogenic agent [10]. However, it soon became clear that virus presence in tumor samples was not related to any carcinogenesis-initiating event, but was rather associated with H-1PV natural tropism for human cancer cells. Indeed, it was demonstrated that H-1PV is able to suppress chemically [11] or virally [12] induced tumors in animal models. Growing evidence was subsequently gathered showing that H-1PV exerts a broad range of anticancer activities in both preclinical cancer models [13] and in phase I/II clinical trials [14,15]. In particular, H-1PV therapeutic potential in hematological malignancies was documented by demonstrating the efficient regression of Burkitt’s lymphomas (BL) in H-1PV-treated Namalwa tumor-bearing animals [16]. H-1PV-induced suppression of viability was observed not only in BL-derived B-cells, but also in malignant T-lymphocyte cell lines derived from acute T-lymphoblastic leukemia and, notably, CTCL. The capacity of CTCL cells to support H-1PV expression and replication and to undergo virus-induced death [17] encouraged us to believe that parvovirotherapy may hold significant potential in CTCL treatment. With the present study, we initiated the assessment of this potential using, in addition to an expanded panel of cell lines, a novel heterotypic CTCL spheroid model that mimics CTCL cell growth in the presence of various tumor microenvironment (TME) components. 

## 2. Materials and Methods

Cells and cell lines. The CTCL-derived cell lines HH (ATCC, CRL-2105™), HuT 78 (ATCC, TIB-161™), SeAx, and MyLa (a kind gift from Dr. Tobias Hein, German Cancer Research Center, Heidelberg) were grown in Roswell Park Memorial Institute (RPMI) 1640 cell culture medium (cat. # R8758, Sigma-Aldrich, Munich, Germany) supplemented with 10% fetal bovine serum (FBS) (catalogue #10500064, ThermoFisher Scientific, Dreieich, Germany), 1000 U/mL penicillin, and 1 mg/mL streptomycin (catalogue #15140122, Life Technologies, Darmstadt, Germany). Human primary healthy skin fibroblasts (phsF) were generously provided by Prof. Dr. rer. nat. Petra Boukamp and Dr. Hans-Jürgen Stark (German Cancer Research Center, Heidelberg, Germany) and cultured in Dulbecco’s modified Eagle’s medium (DMEM) (cat. #41965039, ThermoFisher Scientific, Dreieich, Germany) supplemented with 10% FBS and antibiotics. Human healthy donor naïve pan T-cells (cat. #70024.1) and peripheral blood mononuclear cells (PBMCs) (cat. #70025.3) were purchased from STEMCELL Technologies GmbH (Cologne, Germany) and cultured in ImmunoCult™-XF T Cell Expansion Medium (cat. #100-0956, STEMCELL Technologies GmbH, Cologne, Germany) or RPMI 1640 supplemented with FBS and antibiotics, respectively. Pan T-cell activation was triggered by primary cell culture treatment with the ImmunoCult™ Human CD3/CD28/CD2 T Cell Activator (cat. #10970, STEMCELL Technologies GmbH, Cologne, Germany) following STEMCELL recommendations. PBMC activation was performed by stimulating the cells with one µg phytohemagglutinin (PHA) (cat. #11082132001, Sigma-Aldrich, Taufkirchen, Germany) for 24 h. The spontaneously transformed non-tumorigenic human epidermal keratinocyte cell line HaCaT was a kind gift from P. Boukamp (German Cancer Research Center, Heidelberg, Germany) and was maintained in DMEM medium (cat. #41966029, ThermoFisher Scientific, Dreieich, Germany). Human umbilical cord vein endothelial (HUVEC) cells (ATCC, CRL-1730™) were grown in Endothelial Cell Growth Medium (cat. #211500, Sigma-Aldrich, Munich, Germany).

Cell immunostaining. CTCL cell suspensions were either infected with H-1PV or left untreated. On days one, two and three post-treatment, infected and control suspensions were collected, centrifuged, and resuspended in phosphate buffered saline. Twenty µL cell suspension per well was air-dried on multiwell slides (cat. #6311371, VWR International, Bruchsal, Germany), and slides were processed in the standard manner for immunofluorescence staining. The P2X7 receptor (PA5-77665)- and the CD73 ectonucleotidase (MA5-15537)-specific primary antibodies from ThermoFisher Scientific (Dreieich, Germany) and the H-1PV NS1-specific monoclonal antibody [18], generously provided by Dr. Jürg Nüesch (German Cancer Research Center, Heidelberg, Germany) were used. Images were acquired and analyzed with the motorized inverted Zeiss Cell Observer.Z1 microscope and the Zen 2011 (blue edition) software, respectively. 

Heterotypic CTCL spheroid generation. Heterotypic CTCL spheroids were generated using the methylcellulose-modified hanging-drop method as described elsewhere [19]. Briefly, hanging-drop co-cultures containing 10,000, 30,000 and 20,000 CTCL, phsF (or, alternatively, HaCaT) and HUVEC cells, respectively, per 20 µL drop, were initiated on the lids of 10 cm Falcon™ cell culture dishes (cat. #353003, Corning, Kaiserslautern, Germany). After microscopic verification of spheroid formation (12–24 h post-co-culture initiation), spheroids were transferred, as a single spheroid per well, to 96-well ultra-low attachment (ULA) plates (cat. #650970) from Greiner Bio-One GmbH (Frickenhausen, Germany) and used for immunohistochemical analysis and downstream experiments, as described below.

Heterotypic CTCL spheroid co-cultures with PBMCs. Heterotypic CTCL spheroids (single spheroid per well) were co-cultured with healthy donor PBMCs (20,000 PBMCs per well). Co-cultures were either treated with H-1PV or left untreated (mock). Co-culture supernatants and spheroids were collected on day three after the infection for proinflammatory cytokine release measurement and analysis of spheroid infiltration with immune cells, respectively.

Heterotypic CTCL spheroid immunohistochemical analysis. Spheroid formalin fixation, ROTI^®^Histofix (cat. #P087.1, Carl ROTH GmbH & CoKG, Karlsruhe, Germany) embedding, processing in paraffin blocks, sectioning, and immunofluorescence staining were performed following the standard protocols. Spheroid sections were stained against either H-1PV non-structural protein NS1 (see above) or various cell type-specific markers. In particular, the following antibodies were used: anti-CD3 (Abcam, ab5690, Cambridge, UK), anti-CD4 (Novus Biologicals, NBP1-19371), anti-vimentin (Abcam, ab8978), anti-pancytokeratin (Abcam, ab86734), and anti-CD31 (Abcam, ab182981), in order to identify T-cells, fibroblasts, keratinocytes, and endothelial cells, respectively. Spheroid sections obtained from spheroids that had been co-cultured with PBMCs were stained against cytotoxic T-lymphocyte-specific markers, in particular CD8, using the ab237709 antibody from Abcam. Image acquisition and analysis were performed using a Zeiss Cell Observer.Z1 microscope and the Zen 2011 (blue edition) software (Oberkochen, Germany). 

Wild-type and recombinant H-1PV production. Wild-type (wt) virus stocks were produced and purified by the Biopharmaceutical Production and Development Unit (BP&DU, German Cancer Research Center, Heidelberg, Germany) as described elsewhere [20]. Infectious virus titers were determined using virus plaque assays and expressed as plaque-forming units (PFU) per mL virus stock. H-1PV stocks were serially diluted in serum-free cell culture medium to the desired multiplicity of infection (MOI, PFU per cell). The recombinant defective H-1PV/EGFP virus, in whose genome the sequence coding for the viral capsid proteins was replaced by an enhanced green fluorescent protein (EGFP)-coding sequence, was produced as previously described [21].

H-1PV infection and intracellular detection. Suspension monocultures and spheroids were infected with either the recombinant H-1PV/EGFP (one green fluorescence unit (GFU) per cell) or with the wt virus at various MOIs (0.1–50 PFU per tumor cell). H-1PV uptake and genomic DNA expression were studied by examining the samples for the presence of a positive green fluorescence signal after infection with H-1PV/EGFP. The expression of wtH-1PV NS1 protein was visualized using an NS1-specific monoclonal antibody (see above). Whole wtH-1PV virion detection in spheroid lysates was done using the BL-H1 antibody raised against parvovirus capsids [20].

Kinetic analysis of virus genomic DNA amplification and production of infectious progeny virions. Virus genomic DNA amplification and capacity for plaque formation were quantified as described previously. Briefly, a 141-nt fragment within H-1PV NS1 gene was amplified and detected using a 5′-FAM-MGB-3′ labeled TaqMan probe. Quantitative real-time PCR reaction mix (20 µL) contained 1× Premix Ex Taq™ (TaKaRa, Saint-Germain-en-Laye, France), 0.3 μM labeled NS1-TaqMan™ probe, 0.3 μM primer mix, and 3 µL template. The reaction was run in an Abi Prism 7900 HT Sequence Detection System and data were processed using the SDS 2.1 software (Applied Biosystems, Darmstadt, Germany). Plaque assays were performed using NB-324K cells infected with serial H-1PV dilutions and overlaid with bacto-agar (Becton Dickinson, Heidelberg, Germany). Living cells were stained with 0.02% toluylene red solution (Sigma, Germany) on day four after infection, and plaques were counted on a light box one day later [20].

Cell viability tests. The cytotoxicity induced by wtH-1PV infection of classically grown cell cultures was measured using the CellTiter 96 Aqueous One Solution Cell Proliferation Assay (cat. #G3581, Promega, Walldorf, Germany) according to manufacturer’s instructions, and a Multiskan EX microplate photometer (ThermoFisher Scientific, Dreieich, Germany). Spheroid viability was assessed using the CellTiterGlo 3D Cell Viability Assay (cat. #G9682, Promega, Walldorf, Germany). Luminescence signals were collected using a Mithras2 LB 943 Multimode Reader (Berthold Technologies, Bad Wildbad, Germany). Viability measurements were performed at various time points after infection and data were compared between H-1PV-treated and mock-treated (control) samples.

Live-cell imaging. The effects of H-1PV treatment on CTCL spheroid growth were evaluated using the dead cell tracker dye IncuCyte^®^ Cytotox Red (cat. #4632, Sartorius, Göttingen, Niedersachsen, Germany) and three-day live-cell imaging with an IncuCyte S3 incubator microscope (Essen BioScience, Ann Arbor, MI, USA). Spheroid size and intracellular Cytotox Red dye incorporation were analyzed using the IncuCyte 2019B Rev 2 software. 

Immunogenic cell death (ICD) and proinflammatory cytokine release measurements. Extracellular ATP (eATP) levels in culture supernatants were determined using the RealTime-Glo Extracellular ATP Assay (cat. #GA5012, Promega, Walldorf, Germany) following the manufacturer’s recommendations. Luminescence measurements were collected using a Mithras2 LB 943 Multimode Reader (Berthold Technologies, Bad Wildbad, Germany). Raw luminescence data were normalized to the cell contents and results were displayed as relative luminescence units (RLU) per 10^3^ cells. The extracellular release of high mobility group box 1 (HMGB1), tumor necrosis factor-alpha (TNF-α), interleukin-6 (IL-6), and granulocyte-macrophage colony-stimulating factor (GM-CSF) was analyzed using ELISA kits commercially available from Novus Biologicals (Abingdon, UK) (HMGB1, cat. #NBP2-62766), Bio-Techne (Wiesbaden, Germany) (TNF-α, cat. #NBPP1-183741, and IL-6, cat. #NBP1-89869), and Hoelzel Diagnostika Handels GmbH (Cologne, Germany) (GM-CSF, cat. #ELH-GMCSF-1), according to the manufacturer’s instructions.

Statistics. Experiments were performed in duplicate. Six to twelve replicates were used per treatment condition. Data are presented as mean + SD. 

## 3. Results

### 3.1. CTCL Cells Efficiently Support H-1PV Replication 

**Figure 1:**
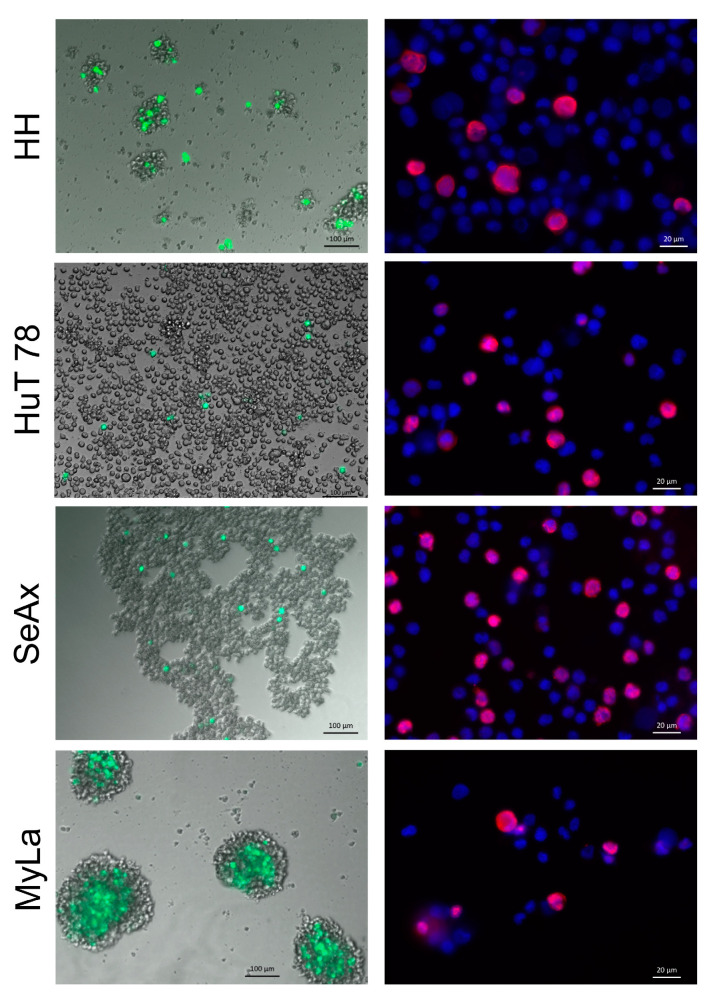
H-1PV entry and genomic DNA expression in CTCL cells. Positive fluorescence signals, indicative of virus P4 promoter activity, NS1 protein synthesis, and NS1-mediated virus P38 promoter activation, were detected on day three after infection with the recombinant H-1PV/EGFP virus (**left** panels, green) in all CTCL cell lines. Accordingly, H-1PV NS1 protein, the major inductor of virus-mediated oncotoxicity, was detected (**right** panels, red) in all cell lines after infection with the wt virus.

H-1PV uptake, non-structural protein expression, genomic DNA amplification, and the assembly and release of infectious progeny virions were studied in classically grown HH, HuT 78, SeAx, and MyLa cultures. The cells were infected with the recombinant H-1PV/EGFP virus and microscopically examined for the generation of a positive fluorescence signal on day three after infection. All CTCL cell lines supported virus entry and promoter activation, as demonstrated by the observation of EGFP expression (Figure 1, left panels) that persisted until at least day ten after infection. Infection with the wt virus led to the detection of abundant H-1PV NS1 expression (Figure 1, right panels) in all cell lines tested. 

H-1PV propagation in CTCL cells was studied through kinetic analysis of virus genomic DNA amplification and production of infectious progeny virions. H-1PV genomic DNA amplification was most pronounced in HH cells, in which a five-log increase in the quantity of viral genomes (vgs) per mL cell lysate was observed between eight and seventy-two hours post infection (Table 1). In H-1PV-infected HuT 78, SeAx, and MyLa cells, an approximately three-log gain was observed. Importantly, de novo synthesized vgs were assembled into infectious progeny virus particles capable of inducing plaque formation, as demonstrated by the progressive increase in the infectious virus titer (PFU per mL cell lysate) (Table 1). Once more, HH cells were the most efficient in supporting H-1PV productive infection, while in SeAx cells only a ten-fold increase in the infectious virus titer was detected on day three after infection. 

Of note, in contrast to CTCL cells, human primary healthy donor pan T-cells supported only very low levels of H-1PV genomic DNA amplification (Supplementary Table S1). This observation was in agreement with the capacity of these cells to allow virus uptake (Supplementary Figure S1), but to resist wtH-1PV-induced cytotoxicity (see below).

### 3.2. CTCL Cells Are Sensitive to H-1PV-Induced Oncolysis 

**Figure 2:**
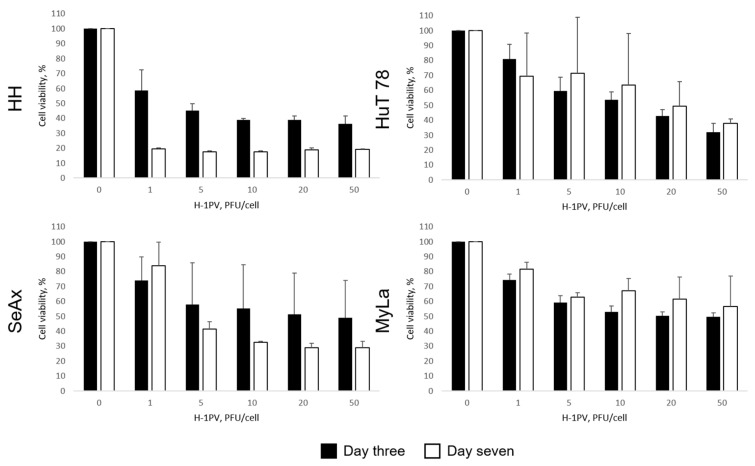
H-1PV-mediated induction of cytotoxicity in CTCL cells. On day three after infection, significant viability suppression was observed in HH, HuT 78, and MyLa cells, while in SeAx cells, in contrast, the 50% TCID could not be reached. On day seven after infection, progression of virus-induced oncolysis was seen in HH and, of note, in SeAx cultures, in contrast to the overgrowth of HuT 78 and MyLa cell fractions, which survived infection on day three.

All four CTCL cell lines displayed, although to a varying extent, significant sensitivity to H-1PV-induced oncolysis. Further to their highest capacity for supporting H-1PV genomic DNA amplification, HH cells exhibited the strongest responsiveness to virus-induced cell killing. In HH cultures, five PFU per cell was sufficient to cause 50% reduction in cell viability on day three after infection (Figure 2, black bars). A quantity of 20 PFU per cell was required to achieve comparable viability suppression in HuT 78 and MyLa cells. In contrast to HH, HuT 78, and MyLa, the 50% tissue culture infectious dose (TCID) in SeAx cells could not be reached on day three after infection. Therefore, based on the 50% TCID values on day three, SeAx cells were considered the most resistant to virus-induced oncolysis (Table 2). However, on day seven after H-1PV treatment (Figure 2, white bars), in HH, but, remarkably, also in SeAx cultures, a higher viability suppression, in comparison with the same virus dose on day three, was induced. In contrast, overgrowth of the fraction of those HuT 78 and MyLa cells that were able to escape the virus or survive infection on day three was observed on day seven.

**Figure 3:**
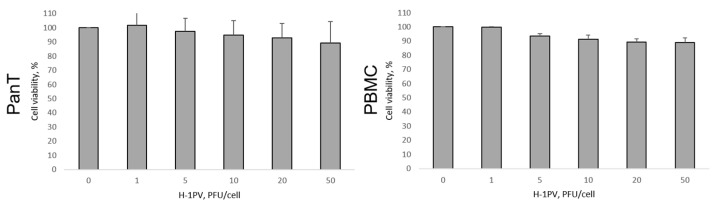
H-1PV innocuousness for human primary healthy donor pan T-lymphocytes (panT) and peripheral blood mononuclear cells (PBMC). H-1PV infection of activated panT and PBMC cells failed to induce any significant toxicity, in contrast to the substantially impaired viability observed on day three in H-1PV-infected CTCL cultures (see above).

In contrast to the substantial cytotoxicity induced in CTCL cells, H-1PV infection was innocuous for control healthy donor pan T-cells (Figure 3, left panel). Only minor, virus dose-independent cytotoxicity was detected in these cells, irrespective of their activation status and in agreement with their low capacity for supporting virus genomic DNA amplification (see above). Furthermore, as previously reported by others [22], the virus failed to exert toxic effects in healthy donor PBMCs (Figure 3, right panel), despite the ability of these cells—similar to pan T-lymphocytes—to allow H-1PV entry (Supplementary Figure S1).

### 3.3. H-1PV-Induced Oncolysis in CTCL Cells Is Associated with the Induction of Extracellular ATP Release 

**Figure 4:**
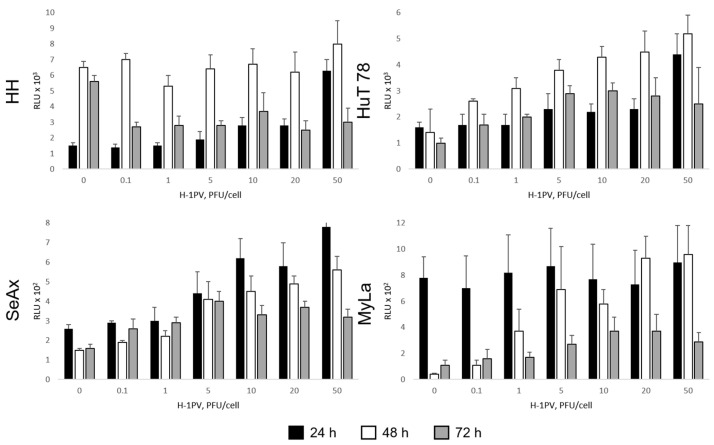
Induction of eATP release in H-1PV-infected CTCL cultures. In HH cells, which are prone to efficient quick oncolysis, increased eATP accumulation was detected twenty-four hours after infection with high (>10 PFU/cell) virus doses. In HuT 78, SeAx, and MyLa cells, which were found to display lower sensitivity to the virus, in comparison with HH, eATP secretion was induced up to seventy-two hours, and peaked forty-eight hours post infection. RLU, relative luminescence unit.

The extracellular release of two recognized soluble markers of ICD, in particular eATP and HMGB1, was measured in virus-infected, in comparison with mock-treated, culture supernatants. In HH cells, increased eATP accumulation (up to four-fold enhancement over the control) was caused by higher virus doses on day one, but not at later time points after infection (Figure 4). In these highly responsive cells, H-1PV-induced oncolysis appeared to be efficient enough to prevent further increase in the induction of ICD signaling. In HuT 78, SeAx, and MyLa cultures, in which higher H-1PV doses or a longer time post infection were required for cell viability to be reduced by 50% (see above), the increase in eATP release peaked forty-eight hours after the infection and was particularly pronounced (twenty-four-fold increase over the control) in MyLa cells. Interestingly, in the absence of virus infection, enhancement of eATP accumulation was observed in HH supernatants between twenty-four and forty-eight hours of culture proliferation. In contrast, we failed to make a similar observation in healthy skin fibroblast, normal endothelial cell and non-tumorigenic keratinocyte cultures. Of note, H-1PV infection of HaCaT keratinocytes, which led to the induction of insignificant cytotoxicity (Supplementary Figure S2a), was accompanied by increased eATP secretion forty-eight hours after the treatment (Supplementary Figure S2b). H-1PV treatment was not associated with the induction of detectable increase in the extracellular HMGB1 release in any of the CTCL cell lines. Notably, abundant HMGB1 amounts (>2000 pg/mL) were detected, irrespective of virus infection, in SeAx culture supernatants, in agreement with the clinical finding that this signaling molecule may be involved in CTCL progression [23].

Next, we established an additional CTCL in vitro model with the aim to examine H-1PV-induced oncolysis in an experimental setting with increased in vivo relevance. To this end, we initiated, to the best of our knowledge, for the first time in cutaneous lymphoma research, hanging-drop CTCL co-cultures with either fibroblasts or, alternatively, keratinocytes, and endothelial cells, in order to generate triple co-culture heterotypic spheroids and mimic the spatial growth of CTCL in the presence of TME.

### 3.4. CTCL Cells Require the Support of Tumor Microenvironment Components in Order to Display a Three-Dimensional Growth Pattern In Vitro 

**Figure 5:**
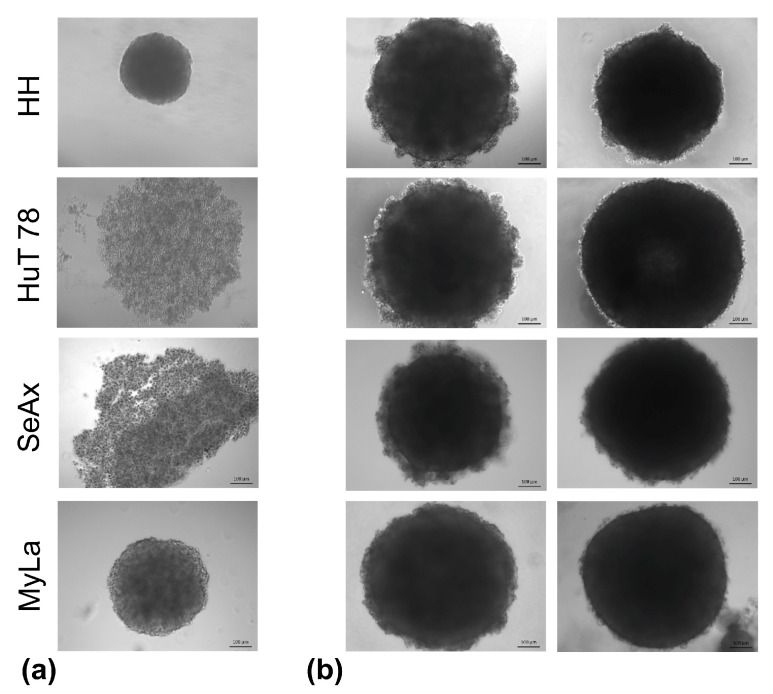
Spheroid formation in hanging-drop CTCL cultures. (**a**) In contrast to HH and MyLa cells, which displayed intrinsic spheroid-building ability, HuT 78 and SeAx cells were only capable of two-dimensional clustering under monoculture conditions; (**b**) under co-culture conditions, keratinocytes (**left** panels) or fibroblasts (**right** panels) provided the CTCL cells with substantial 3D growth support resulting in the reproducible formation of compact, regular-shape spheroids.

When cultured alone in a hanging-drop setting, the CTCL cells displayed low intrinsic capacity for spheroid formation. While HH and MyLa cells were able to build loose spheroids, HuT 78 and SeAx cells were only capable of two-dimensional clustering, with no 3D structure formation (Figure 5a). To overcome this limitation, we enriched the hanging-drop cultures with additional cell types that served two purposes: (i) to provide the CTCL cells with a 3D growth support and (ii) to mimic the in vivo situation, in which various non-CTCL cell types are present in the CTCL microenvironment. In particular, either phsF fibroblasts or HaCaT keratinocytes, and HUVEC endothelial cells were co-cultured with CTCL cells in methylcellulose-reinforced hanging-drop conditions. Both double and triple co-culture (Figure 5b) spheroids were reproducibly generated and shown to be regular-shape, compact, and agitation-resistant. Spheroid viability and proliferation were examined by microscopic observation, and confirmed in CellTiterGlo 3D viability assays and live-cell imaging analysis. An average brightfield object area gain was documented in all types of spheroids (Supplementary Figure S3a). As stated below, the proliferation of CTCL cells more particularly contributed to the spheroid growth. Only viable proliferating spheroids were used in downstream experiments.

### 3.5. Heterotypic CTCL Spheroids Typically Exhibit a “Core-Periphery” Structure

**Figure 6:**
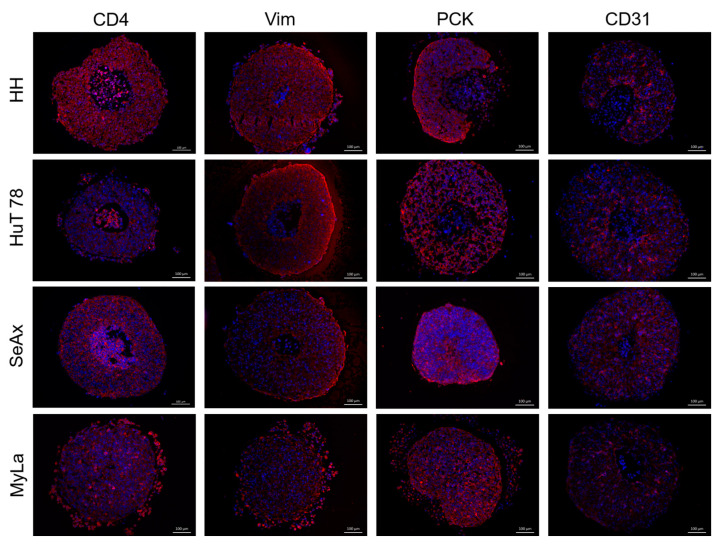
Immunofluorescence detection of cell type-specific markers in heterotypic CTCL spheroids. Immunofluorescence staining against CD4, vimentin (Vim), pancytokeratin (PCK), and CD31 (red) verified the incorporation into the heterotypic spheroid of lymphoma, fibroblast, keratinocyte, and endothelial cells, respectively.

**Figure 7:**
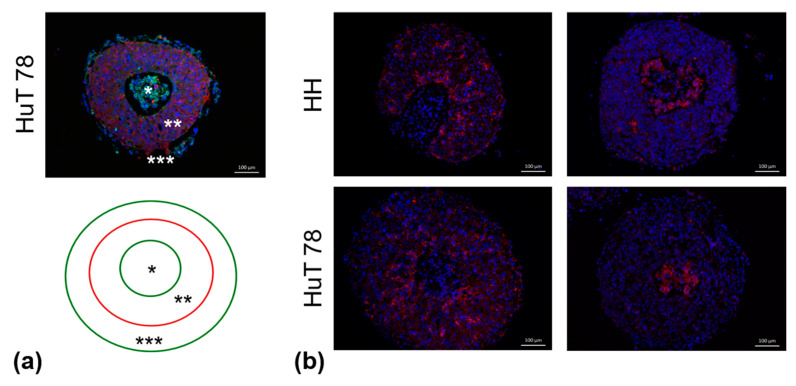
Heterotypic CTCL spheroid structure. (**a**) The characteristic “core-periphery” structure displayed by all types of heterotypic CTCL spheroids is illustrated by the immunofluorescence labeling of keratinocyte-containing HuT 78 spheroid sections. The typical heterotypic CTCL spheroid consisted of: (i) CTCL core (*, CD4, green), (ii) TME zone (**, pancytokeratin, red), and (iii) an invasive CTCL periphery (***, CD4, green). (**b**) In fibroblast-containing HH and HuT 78 spheroids (**left** panels), the endothelial cells (CD31, red) populated the entire TME zone. In contrast, in the keratinocyte-containing counterparts (**right** panels), the CD31+ cells were shown to cluster around the CTCL core.

The formation of rigid, regular-shape spheroids approximately twenty-four hours after the initiation of the hanging-drop co-cultures was verified by microscopic observation. Spheroids were collected, processed and subjected to immunofluorescence labeling of various cell type-specific markers as described in the Materials and Methods section. Immunofluorescence labeling confirmed the integration of all hanging-drop co-culture partners into the individual spheroid structure (Figure 6) and revealed that all types of CTCL spheroids consistently displayed a characteristic “core-periphery” structure. Typically, a spheroid section was characterized by the presence of (i) CTCL core, (ii) TME zone composed of fibroblasts (or keratinocytes) and endothelial cells, and (iii) CTCL periphery. This peripheral zone mostly comprised quickly proliferating lymphoma cells and represented the invasive front of the spheroid (Figure 7a and Supplementary Figure S3b). In fibroblast-containing HH and HuT 78 spheroids, the endothelial cells were found to populate the entire TME zone (Figure 7b, left panels). Interestingly, when fibroblasts in the triple co-culture were replaced by keratinocytes, the endothelial cells in the resulting spheroids were shown to cluster around the CTCL core (Figure 7b, right panels). In addition, double immunofluorescence labeling revealed some peculiar protein expression patterns. On one hand, CTCL cells, in particular those located at the invasive periphery of the spheroid, unexpectedly displayed vimentin and von Willebrand factor (VWF) positivity (Supplementary Figure S4a), typically found in fibroblasts and endothelial cells, respectively. On the other hand, CTCL cells lacked the expression of the C-C chemokine receptor 4 (CCR4), a molecule considered a potential disease marker and therapeutic target [24,25]. CCR4 expression was nevertheless detected in pancytokeratin-positive cells within the TME zone of the spheroids and was therefore assigned to the keratinocyte component (Supplementary Figure S4b). 

### 3.6. H-1PV Efficiently Infects Heterotypic CTCL Spheroids and Induces Inhibition of Their Growth and Viability

**Figure 8:**
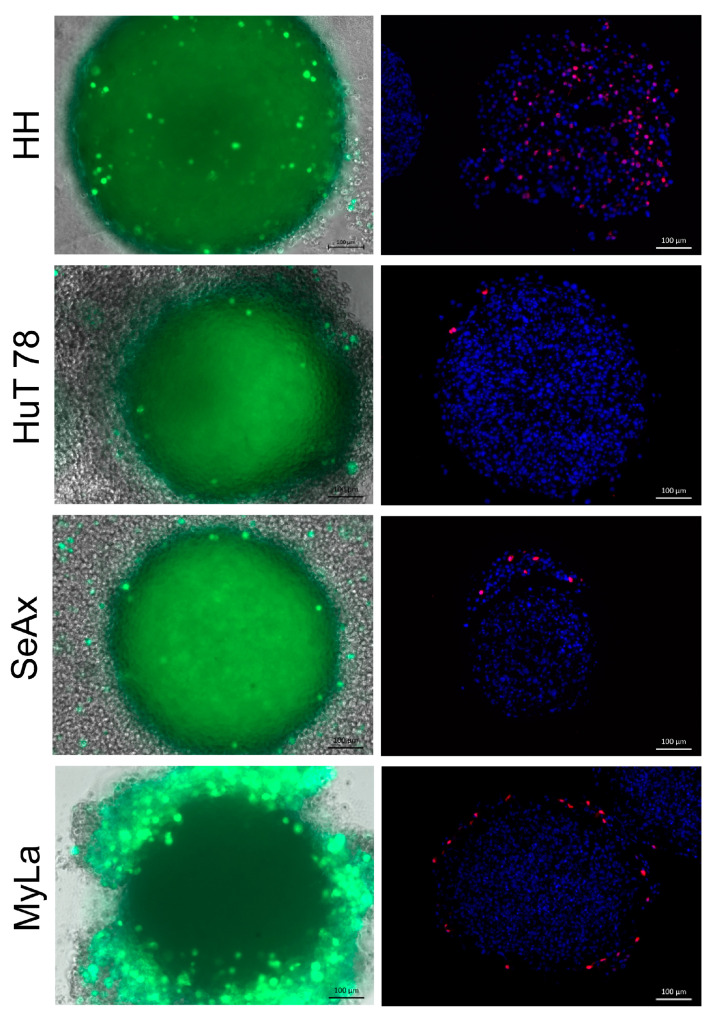
Reporter and viral gene expression in H-1PV-infected heterotypic CTCL spheroids. Reporter EGFP (green, **left** panels) and NS1 (red, **right** panels) protein expression generated positive immunofluorescence signals detected three days after spheroid infection with recombinant H-1PV/EGFP or wtH-1PV, respectively.

All types of heterotypic CTCL spheroids were able to support H-1PV infection and replication. Indeed, reporter gene expression was observed after spheroid infection with the recombinant H-1PV/EGFP virus (Figure 8, left panels). Spheroid treatment with wtH-1PV resulted in virus genomic DNA amplification (Supplementary Table S2) and NS1 protein synthesis (Figure 8, right panels). Whole H-1PV virions were detected in spheroid lysates on day three after infection (Supplementary Figure S5). In contrast, control HUVEC spheroids were unable to support H-1PV propagation, as demonstrated by the ten-fold decrease in the amount of input vg per mL spheroid lysate seventy-two hours after the infection (Supplementary Table S2). 

**Figure 9:**
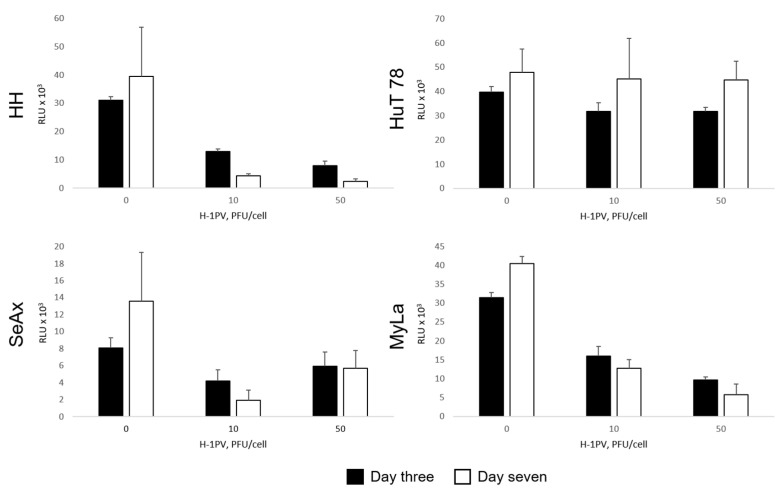
H-1PV-induced suppression of heterotypic CTCL spheroid viability. Virus treatment of HH, SeAx, and MyLa spheroids led to progressive reduction in spheroid viability, in comparison with mock-treated controls, which ensued from day three to day seven. In HuT 78 spheroids, the moderate H-1PV-induced cytotoxicity detected on day three was not detectable on day seven. RLU, relative luminescence unit.

**Figure 10:**
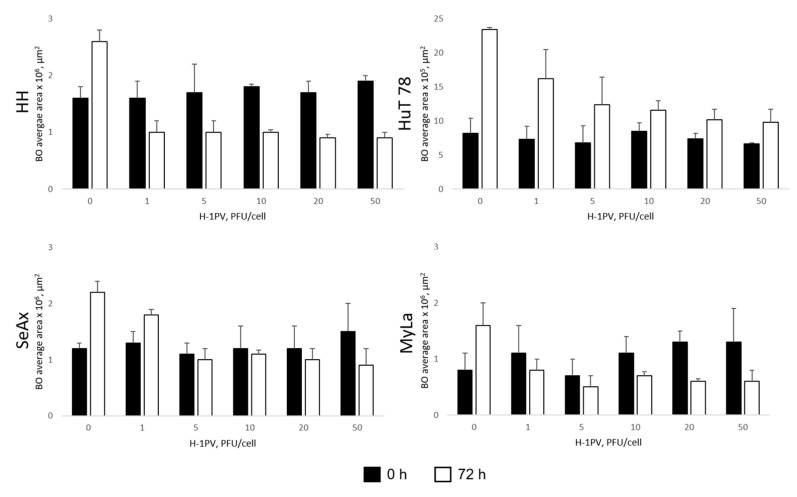
H-1PV-induced shrinkage of heterotypic CTCL spheroids. The spheroids were treated, or not, with H-1PV and the reduction in brightfield object (BO) average area was evaluated using live-cell imaging analysis. Data obtained at 0 h (spheroid treatment and start of imaging) and 72 h are displayed and show significant shrinkage of all spheroids on day three after infection.

**Figure 11:**
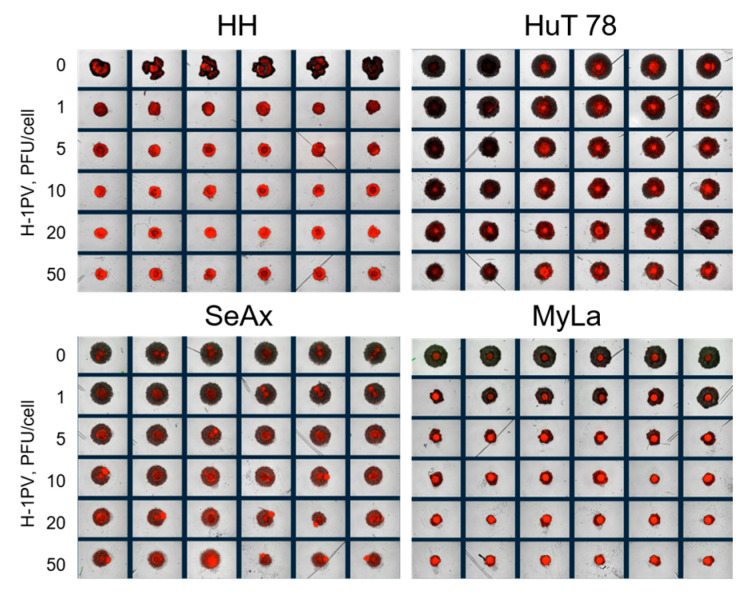
H-1PV-induced cytotoxicity in heterotypic CTCL spheroids. On day three after spheroid treatment with H-1PV (n = 6 per treatment condition), virus dose-dependent increase in the uptake of the dead cell tracker IncuCyte^®^ Cytotox Red Dye, in association with significant spheroid shrinkage, was observed.

H-1PV treatment of CTCL spheroids led to significant suppression of spheroid viability (Figure 9). In HH, SeAx, and MyLa spheroids, virus-induced cytotoxic effects progressed until at least day seven after infection. In HuT 78 spheroids, similar to the observation made in HuT 78 suspension monocultures (see above), virus-induced oncolysis was unable to subdue spheroid growth, as demonstrated by the increase in the viable cell fraction on day seven (white bars), in comparison with day three (black bars). Spheroid shrinkage was nevertheless observed in all, including HuT 78, spheroids seventy-two hours after virus infection (Figure 10). Indeed, virus-induced intoxication on day three could be visualized through the virus dose-dependent enhancement of the dead cell tracker IncuCyte^®^ Cytotox Red Dye incorporation in all spheroids (Figure 11). 

### 3.7. H-1PV Treatment of Keratinocyte-Containing CTCL Spheroid Co-Cultures with PBMCs Induces Increased Spheroid Infiltration with CD8+ T-Lymphocytes

**Figure 12:**
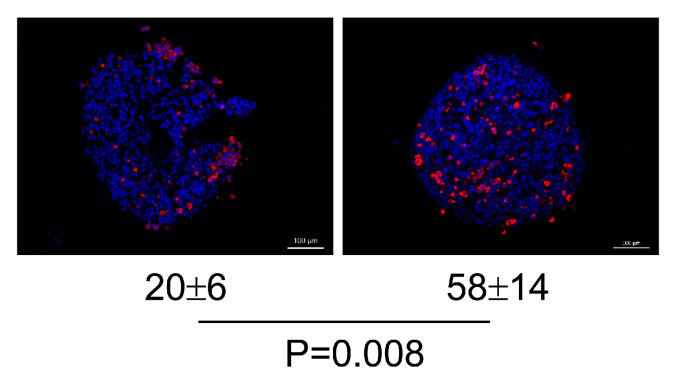
Spheroid infiltration with CD8+ cells in co-cultures of keratinocyte-containing MyLa spheroids and PBMCs. H-1PV treatment (**right** panel) of the co-culture enhanced the spontaneous (**left** panel) spheroid infiltration with cytotoxic T-lymphocytes (CD8, red). Mean CD8+ cell counts per 500 cells were compared between H-1PV-infected and mock-treated co-cultures.

The peculiar development of CTCL in the upper skin layer raises the question of the impact of keratinocytes on the fate of this disease. It is indeed known that by secreting cyto- and chemokines, keratinocytes can recruit, activate, and regulate immune cells [26]. This prompted us to co-culture keratinocyte-containing MF spheroids and healthy donor PBMCs, and to determine whether proinflammatory cytokines were released under these conditions and spheroids were infiltrated with specific immune cells. Since the anticancer activity of OVs proved to result, at least in part, from their ability to boost antitumor immune responses [27], the effects of H-1PV infection of these co-cultures on the above parameters were also investigated. We observed that keratinocyte-containing MyLa co-cultures produced a basal level of TNF-α, which increased upon H-1PV infection (Supplementary Figure S6). Interestingly, this TNF-α induction was not detected in supernatants from fibroblast-containing MyLa spheroid co-cultures, in support of the possible contribution of keratinocytes to the production of this cytokine [26,28]. However, it has to be noted that, within the limited sample size used in our pilot studies, TNF-α induction did not reach statistical significance and further investigations are worth conducting in order to substantiate this finding. In contrast, high basal levels (>100 pg/mL) of IL-6 are known to be involved in CTCL pathogenesis [29], but no changes in both IL-6 and GM-CSF levels were observed after co-culture infection with H-1PV. In addition, keratinocyte-containing MyLa spheroids were found to undergo a spontaneous infiltration with CD8+ cells (Figure 12, left panels). This infiltration was increased upon infection of co-cultures with H-1PV (Figure 12, right panels), showing that, in an admittedly artificial context still mimicking the in situ situation, heterotypic CTCL spheroids respond to H-1PV infection by enhancing the recruitment of cytotoxic T-lymphocytes.

## 4. Discussion

Oncolytic virotherapy, an emerging cancer treatment modality, is recently attracting growing awareness. OVs, with their capacity for both inducing selective cancer cell death and triggering antitumor immune responses, hold significant potential for becoming next-generation cancer immunotherapeutics [27,30]. However, the oncolytic virotherapy approach currently remains underappreciated in hematological malignancies of T-cell origin, in particular, in CTCL. Promising anticancer effects of an oncolytic measles virus have been reported in vitro and in CTCL patients [7,8]. Our literature search for other OV applications in cutaneous lymphoma failed to produce any further results, thus emphasizing the need for oncolytic virotherapy consideration in the context of CTCL. The aim of the present study was to conduct—to the best of our knowledge, for the first time in CTCL research—a preclinical investigation of H-1PV, an oncolytic protoparvovirus, and its therapeutic potential in in vitro models of the disease. 

**Figure A1:**
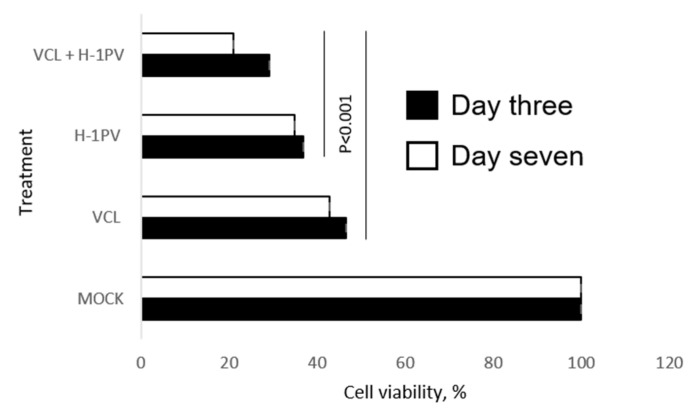
Improved SeAx cell killing by combined H-1PV infection and Bcl-2 inhibition. Venetoclax (VCL, 100 nM)-mediated Bcl-2 inhibition combined with H-1PV infection (10 PFU/cell) of Bcl-2-overexpressing SeAx cells led to increased suppression of cell viability on both day three and day seven, in comparison with either virus or VCL treatment alone.

We used a panel of four commercially available human CTCL-derived cell lines, in all of which we demonstrated efficient H-1PV entry, genomic DNA amplification, protein expression, assembly of infectious progeny virus particles, and, ultimately, oncolysis. The individual sensitivity to the virus varied among the cell lines and suggested a possible link with their Bcl-2 expression status. Among the cell lines, HH cells displayed the highest capacity for virus propagation and most efficiently succumbed to oncolysis. These cells were indeed reported to be Bcl-2-negative. In contrast, SeAx cells, in which the 50% TCID of the virus could not be determined on day three after infection, were shown to be Bcl-2-overexpressing [31]. Further to the above, HuT 78 and MyLa cells, which are known to express Bcl-2, however, at significantly lower levels than CTCL patient-derived primary samples [32] and SeAx, were indeed less sensitive to the virus than HH cells, but more responsive than SeAx cells on day three after infection. In the latter cells, combining Bcl-2 inhibition with H-1PV treatment resulted in enhanced cytotoxicity (Appendix A, Figure A1), raising the possibility for exploring this approach in order to achieve increased efficiency of CTCL cell eradication. 

In contrast to CTCL cells, human healthy donor naïve pan T-lymphocytes and PBMCs, whereas allowing virus entry, were non-responsive to H-1PV-induced cytotoxicity. H-1PV innocuousness for non-malignant peripheral blood immune cells was further supported by our finding that normal pan T-cells lacked the capacity for supporting H-1PV propagation. As concerns PBMCs, abortion of the virus replicative cycle has been reported by others [22,33]. Moreover, we demonstrated H-1PV preference for malignant CTCL cells over non-malignant cells from the CTCL microenvironment. In particular, we observed lack of virus-induced cytotoxicity in human primary healthy skin fibroblasts (Supplementary Figure S7) and in non-tumorigenic keratinocytes. As regards normal human umbilical vein endothelial cells, which were also part of our heterotypic spheroid model, virus uptake has been shown to result in abortive infection [34] and no H-1PV-induced viability suppression could be documented (Supplementary Figure S8). 

**Figure 13:**
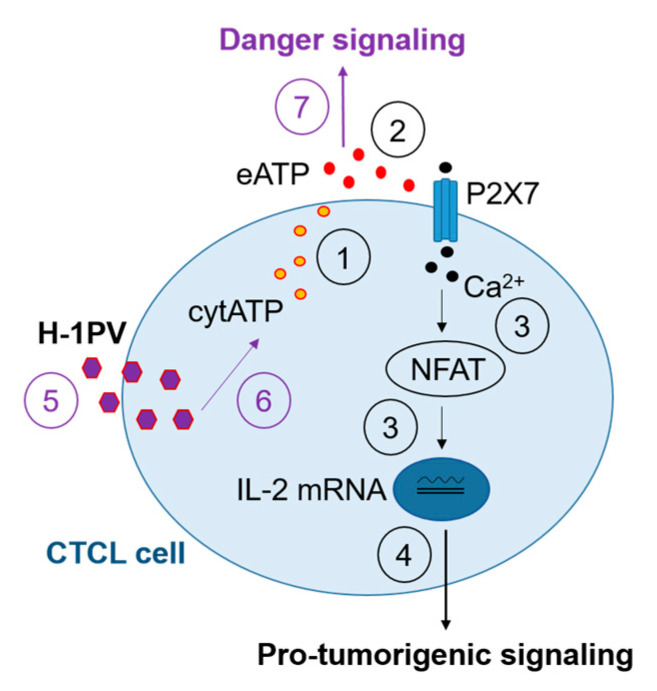
The multifaceted role of extracellular ATP in CTCL biology. CTCL cells release cytosolic ATP (cytATP) into the extracellular space (1). Extracellular ATP (eATP) may activate cellular purinergic P2X7 receptors (2), leading to Ca^2+^ influx, NFAT activation, IL-2 transcription (3), and, ultimately, CTCL cell growth and proliferation (4). On the other hand, H-1PV infection of CTCL cells (5) may additionally trigger eATP secretion, resulting in the induction of danger signaling in the CTCL TME (7).

**Figure A2:**
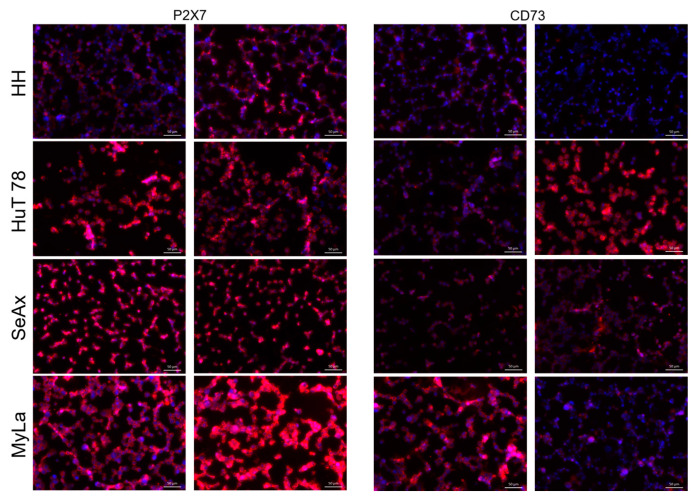
Surface P2X7 purinergic receptor and CD73 ectonucleotidase expression in H-1PV-infected (**right** panels) versus mock-infected (**left** panels) CTCL cells. In HH and MyLa cells, both P2X7 upregulation and CD73 downregulation was induced by H-1PV infection. In contrast, marked CD73 upregulation was observed in infected HuT 78 cells.

H-1PV-induced CTCL cell death was not associated with the release of HMGB1, an ICD consensus marker, contrary, for example, to H-1PV-infected pancreatic ductal adenocarcinoma cells, whose killing process was shown to be consistently accompanied by HMGB1 secretion [35]. Contrary to HMGB1, extracellular release of ATP was triggered by the virus twenty-four to seventy-two hours after infection in all CTCL cell lines. This finding was confined to CTCL cells, as we failed to make a similar observation in H-1PV-infected healthy skin fibroblasts or HUVEC cells. However, virus uptake by keratinocytes resulted in significant virus dose-dependent eATP induction. In light of the well-recognized role that keratinocytes play in innate immunity [36], the latter finding may hint at the potential of these cells to reshape (through eATP-mediated danger signaling) the CTCL microenvironment upon H-1PV treatment of a CTCL lesion in vivo. Interestingly, eATP accumulation was found to be an intrinsic feature of uninfected proliferating HH cultures, but not of normal/non-tumorigenic fibroblasts, keratinocytes, or endothelial cells. Indeed, it has long been established that eATP secreted by cancer cells may act as driving stimulus for their proliferation, migration, and invasion [37]. However, to the best of our knowledge, the role of this molecule in CTCL biology in particular has not been reported so far. Our observations in HH cells led us to hypothesize that, similar to other types of hematological and non-hematological cancers [38,39], at least some CTCL tumors may use eATP to activate purinergic receptor calcium signaling and stimulate their own growth and proliferation. Indeed, P2X7 or calcineurin (NFAT) inhibition reduced the viability of CTCL cultures, as already demonstrated for B-cell leukemia cells [38], and negatively interfered with H-1PV-induced cytotoxicity (A.A., unpublished observations), suggesting that P2X7-mediated signaling may favorably contribute to the efficiency of H-1PV-induced oncolysis in CTCL cells. We indeed observed that in HH and MyLa cells, the ATP-dephosphorylating ectonucleotidase CD73 was downregulated, and P2X7 was, in contrast, upregulated by H-1PV infection (Figure A2). Virus-mediated modulation of purinergic receptor signaling may be a specific strategy used by H-1PV in CTCL in order to condition the intracellular milieu to the benefit of successful virus propagation. Notably, significant CD73 upregulation was induced by the virus twenty-four hours after the infection of HuT 78 cells. Whether this ectonucleotidase upregulation may be relevant to the decreased efficiency of oncolysis observed at later time points after infection in HuT 78 cells remains to be proven. While further investigations are necessary to elucidate the involvement of the eATP-P2X7 signaling axis in H-1PV-induced CTCL oncolysis, our current findings suggest that eATP released by CTCL cells may play two opposing roles in CTCL biology. On the one hand, it may act as an autocrine pro-tumorigenic stimulus. On the other hand, it may function as an immunogenic danger-signaling molecule capable of alerting the immune system against H-1PV-infected CTCL cells. The balance between these two effects may depend on eATP levels, and thereby on H-1PV treatment (Figure 13). 

The capacity of H-1PV to induce cytotoxic effects in CTCL cells grown in standard culture conditions was preserved in a heterotypic spheroid model. The establishment of the latter model aimed at achieving a closer relevance to the in vivo CTCL situation. By co-culturing various cell types, in particular, CTCL cells, fibroblasts (or, alternatively, keratinocytes), and endothelial cells, we generated triple co-culture spheroids that mimicked the spatial growth of CTCL cells in the presence of non-CTCL cell types. All spheroids displayed a characteristic “core-periphery” structure, with a large TME zone enclosing a lymphoma core. Spheroid growth ensued through the expansion of its invasive front that was formed by quickly proliferating CTCL cells. Immunohistochemical analysis of the spheroids, in addition to identifying the individual cell types in the heterotypic system, produced some intriguing findings that might make a novel contribution to the understanding of CTCL biology. We consistently observed dependence of endothelial cell distribution on TME cellular composition. For example, in fibroblast-containing spheroids, the endothelial cells were spread within the entire TME zone. In contrast, in keratinocyte-containing spheroids, the endothelial cells displayed a different distribution pattern and were found clustered around the lymphoma core. This observation raises the intriguing question of whether keratinocytes might have impact on the crosstalk between CTCL and endothelial cells. We also observed that, depending on their microenvironment, CTCL cells might exhibit some peculiar protein expression patterns. In particular, vimentin expression was detected in CTCL cells, interestingly, only in the presence of endothelial cells, i.e., in triple but not in double (A.A., unpublished observations) co-culture spheroids. It has long been established that various cancer cells may overexpress vimentin in order to facilitate epithelial-mesenchymal transition [40] and enhance metastatic cell growth [41]. High intratumoral expression of vimentin was shown to be a predictive marker of histological transformation in follicular lymphoma patients [42]. Vimentin was also identified as a mediator of drug-resistant phenotype in diffuse large B-cell lymphoma [43]. However, to the best of our knowledge, vimentin expression in CTCL has not yet been reported in the published literature. Our observations suggest that the crosstalk between endothelial cells and CTCL cells might trigger vimentin expression in the latter cells, for example, in order to promote their adhesion and transendothelial migration [44]. Further to the above, VWF expression, typically seen in vascular endothelial cells, but found to be acquired de novo and confer increased metastatic activity to some cancer cells [45], was also detected in CTCL cells in triple co-culture spheroids. We therefore believe that elucidating the role of vimentin and VWF expression in CTCL biology may open up new prospects for the development of novel targeted therapies against this disease. Last but not least, we also examined CCR4 expression in triple co-culture CTCL spheroids. CTCL cells have been demonstrated to often overexpress this chemokine receptor [24]. CCR4 is considered a reliable CTCL-specific marker and, therefore, a promising therapeutic target [25]. Surprisingly, we failed to detect any CTCL-associated CCR4 expression. Instead, substantial CCR4 expression was observed, associated with pancytokeratin-positive cells, in the TME zone of the spheroids. Expression of CCR4 by epidermal keratinocytes has been indeed shown and found to be involved in cutaneous immune reactions [46]. Whether keratinocyte infection with H-1PV (or keratinocyte crosstalk with H-1PV-infected CTCL cells) may alter their CCR4 expression pattern and thereby modulate the CTCL microenvironment remains to be elucidated. Ongoing studies in our laboratory aim to shed light on this matter. 

H-1PV treatment of CTCL spheroids induced their efficient viability reduction and growth suppression. Expression of NS1 protein, the major effector of virus-mediated cytotoxicity, was consistently observed in all infected spheroids. Live-cell imaging demonstrated increasing virus dose-dependent dead cell tracker incorporation, indicative of ongoing oncolysis. In CTCL spheroid co-cultures with PBMCs, H-1PV treatment enhanced the spontaneous spheroid infiltration with cytotoxic T-cells, in agreement with the clinical findings made in parvovirus-treated glioblastoma and pancreatic cancer patients [14,15]. Interestingly, this observation was made only in keratinocyte-, but not in fibroblast-containing spheroids, hinting once more at keratinocytes as key players in CTCL pathogenesis. 

## 5. Conclusions

Altogether, our data demonstrate that the oncolytic parvovirus H-1PV has the potential to induce efficient oncolysis in CTCL cells. This virus-mediated CTCL cell death is (i) not compromised by Bcl-2 protein overexpression; (ii) selective, as no virus-induced killing is observed in control cells; and (iii) associated with the induction of the inflammatory mediator eATP. Accumulation of the latter molecule is, in addition, detected during the proliferation of uninfected CTCL cultures, raising the hypothesis that some CTCL cells may use an eATP-mediated signaling mechanism to stimulate their malignant growth.

H-1PV treatment induces loss of viability and shrinkage in heterotypic CTCL spheroids, a novel in vivo-relevant in vitro CTCL model that reconstitutes the presence of the CTCL microenvironment. Infection of CTCL spheroid co-cultures with PBMCs leads to the induction of enhanced spheroid infiltration with cytotoxic T-lymphocytes.

In conclusion, these first preclinical studies strongly suggest that oncolytic virotherapy by means of an oncolytic parvovirus represents a novel approach that may open up new prospects in the development of innovative CTCL treatments.

## Figures and Tables

**Table 1:** Virus genomic DNA amplification and production of infectious progeny virions in H-1PV-infected CTCL cells.

Cell Line	Hours p.i.	Vg/mL CL	Fold Increase	PFU/mL CL	Fold Increase
HH	8	5E6	2E5	4E4	2E4
	24	3E10		3E5	
	48	2E12		4E8	
	72	1E12		6E8	
HuT 78	8	7E7	9E2	6E4	2E2
	24	2E9		2E5	
	48	1E10		2E6	
	72	6E10		1E7	
SeAx	8	4E7	5E2	5E4	1E1
	24	2E9		7E4	
	48	4E10		1E5	
	72	2E10		3E5	
MyLa	8	9E7	2E3	4E4	1E2
	24	1E10		2E5	
	48	7E10		2E6	
	72	2E11		2E6	

P.i., post infection; Vg, viral genome; CL, cell lysate; PFU, plaque-forming unit.

**Table 2:** H-1PV TCID 50% for CTCL cell lines as a function of time post infection.

50% TCID, PFU/Cell			
Cell Line	Day 3	Trend	Day 7
HH	5	↓	<1
HuT 78	20	↑	≥20
SeAx	>50	↓	1–5
MyLa	20	↑	>50

TCID, tissue culture infectious dose; PFU, plaque-forming unit; down arrow, decrease; up arrow, increase.

## Data Availability

The original contributions presented in the study are included in the article/Supplementary Materials, further inquiries can be directed to the corresponding author.

## Supplementary Materials

The following supporting information can be downloaded at: https://www.mdpi.com/article/10.3390/cancers16152711/s1, Figure S1: H-1PV entry and genomic DNA expression in control non-CTCL peripheral blood cells. Figure S2: H-1PV-induced cytotoxicity and extracellular ATP (eATP) release in HaCaT keratinocytes; Figure S3: Heterotypic CTCL spheroid growth and proliferation; Figure S4: Vimentin, von Willebrand factor and CCR4 patterns of expression in heterotypic CTCL spheroids. Figure S5: Detection of H-1PV virions in cell lysates obtained from virus-treated heterotypic CTCL spheroids; Figure S6: Tumor necrosis factor-alpha release in co-cultures of keratinocyte-containing MyLa spheroids and healthy donor PBMCs; Figure S7: H-1PV innocuousness for human primary healthy skin fibroblasts; Figure S8: H-1PV innocuousness for human umbilical vein endothelial (HUVEC) cells; Table S1: Virus genomic DNA amplification (viral genomes per mL cell lysate) in H-1PV-infected human primary healthy donor naïve pan T cells; Table S2: Virus genomic DNA amplification in H-1PV-infected heterotypic CTCL spheroids.
