# Supplementary material for: H-1 Parvovirus-Induced Oncolysis and Tumor Microenvironment Immune Modulation in a Novel Heterotypic Spheroid Model of Cutaneous T-Cell Lymphoma
Source: Cancers (Basel). 2024 Jul 30;16(15):2711. doi: 10.3390/cancers16152711 (PMC11311363; doi:10.3390/cancers16152711)
Supplement: Supplementary file 1 [file cancers-16-02711-s001.zip › cancers-3099318-supplementary.pdf]

Supplementary Table S1. Virus genomic DNA amplification (viral genomes per ml cell lysate) in H-1PV-infected human primary healthy donor naïve pan T cells.

Control	Hours post infection	Non-activated	Activated
Healthy donor naïve pan T	24h	2E7	2E7
	48h	4E6	5E6
	72h	1E7	4E6

Supplementary Table S2. Virus genomic DNA amplification in H-1PV-infected heterotypic CTCL spheroids.

CTCL	Hours post infection	Vg/ml SL
HH	48	7E8
	72	3E9
	144	3E9
HuT 78	48	5E7
	72	1E9
	144	2E9
SeAx	48	4E4
	72	5E7
	144	5E7
MyLa	48	7E4
	72	5E7
	144	5E7
**Control**	**Hours post infection**	**Vg/ml spheroid lysate**
HUVEC	48	3E7
	72	3E6
	144	3E6

Vg, viral genomes; SL, spheroid lysate

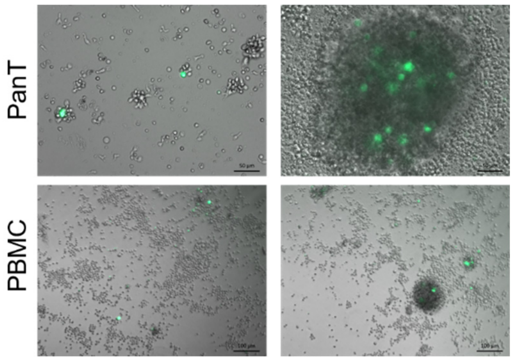

Supplementary Figure S1. H-1PV entry and genomic DNA expression in control non-CTCL peripheral blood cells. Primary healthy donor naïve pan T lymphocytes (PanT) and PBMCs were either activated (right panels), or not (left panels), by treatment with the ImmunoCult™ Human CD3/CD28/CD2 T Cell Activator or 1 μg PHA, respectively. Both non-activated and activated cells were able to support virus entry and genomic DNA expression, as demonstrated by the detection of a positive fluorescence signal (green) after cell infection with the recombinant H-1PV/EGFP virus. However, infection of these cells with the replication-competent wtH-1PV did not lead to the induction of any significant cytotoxicity, in contrast to the pronounced viability reduction observed in wt virus-treated CTCL cultures (see main text).

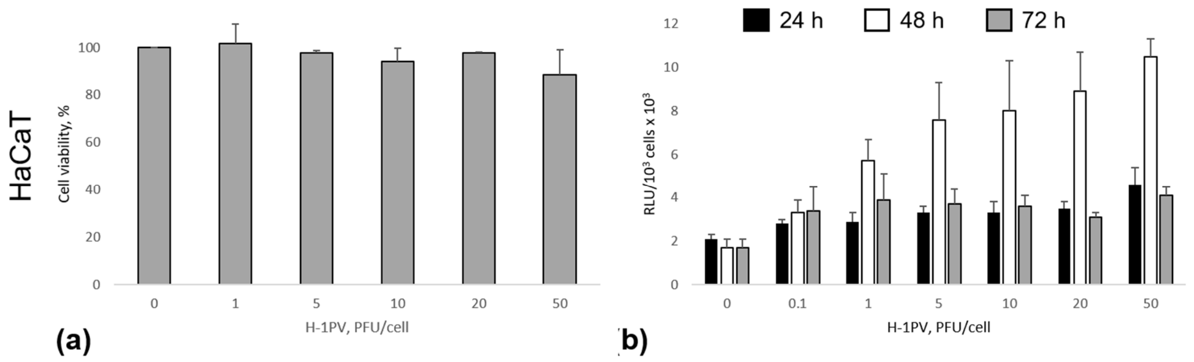

Supplementary Figure S2. H-1PV-induced cytotoxicity and extracellular ATP (eATP) release in HaCaT keratinocytes. (**a**) H-1PV infection of this non-tumorigenic spontaneously transformed keratinocyte cell line resulted in insignificant suppression of cell viability on day three. (**b**) Significantly increased eATP secretion, in comparison with mock-infected controls, was triggered by the virus forty-eight hours after HaCaT culture treatment. RLU, relative luminescence units.

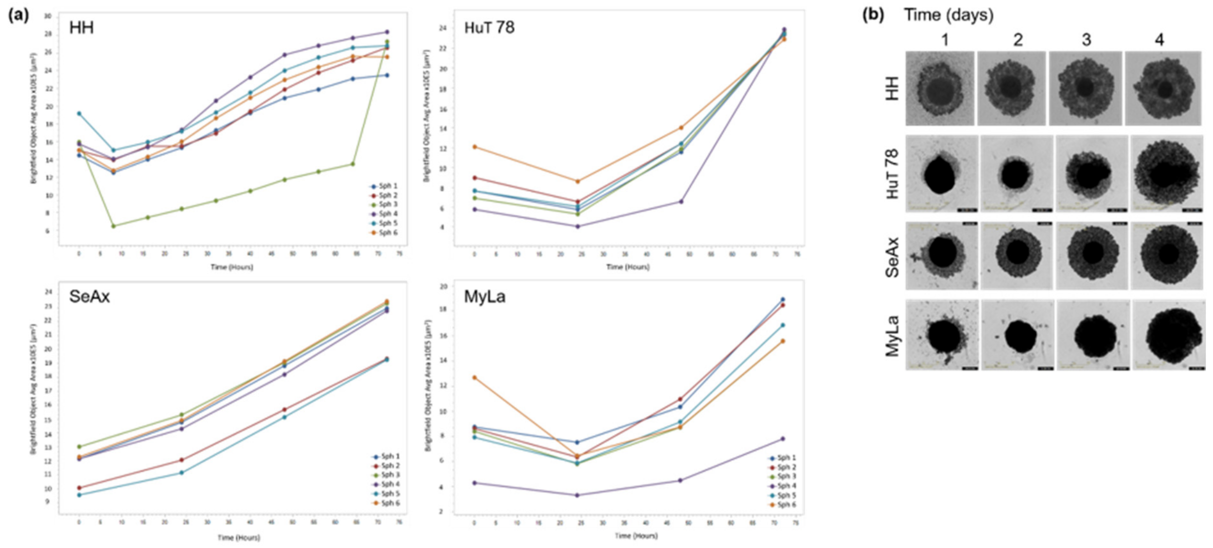

Supplementary Figure S3. Heterotypic CTCL spheroid growth and proliferation. (**a**) Average brightfield object area (μm²) gain was detected between 0 h (spheroid formation and start of live-cell imaging) and 72 h, in both fibroblast-containing (illustrated on the figure) and keratinocyte-containing triple co-culture CTCL spheroids. (**b**) Live-cell imaging of growing spheroids demonstrating the enlargement of the peripheral zone composed of proliferating lymphoma cells.

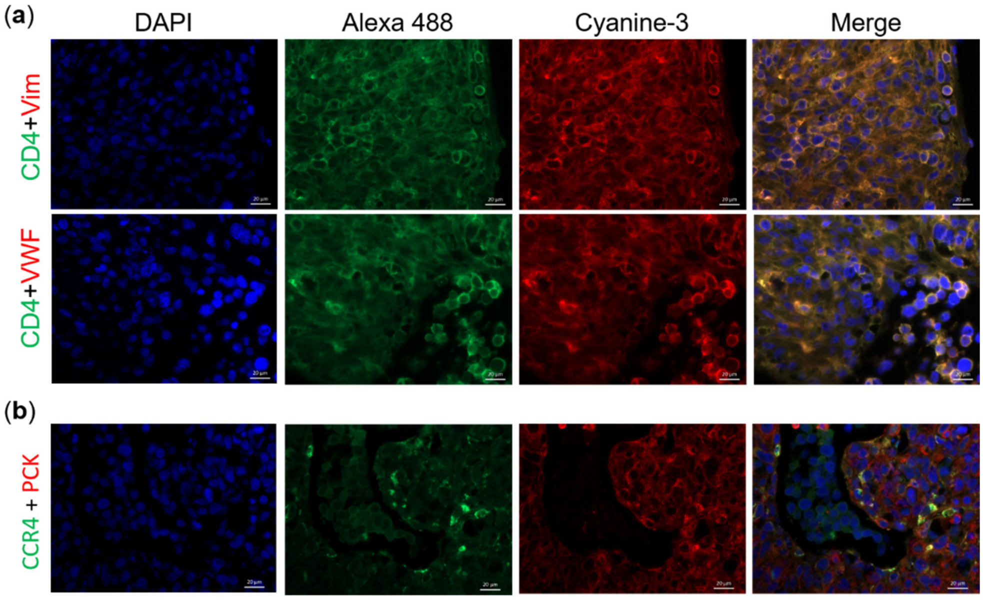

Supplementary Figure S4. Vimentin, von Willebrand factor and CCR4 patterns of expression in heterotypic CTCL spheroids. Double immunofluorescence labeling of heterotypic CTCL spheroid sections (illustrated for HuT 78 spheroids) revealed (**a**) vimentin (Vim, red, upper panels) and von Willebrand factor (VWF, red, lower panels) expression in CD4-positive (green) CTCL cells located in the peripheral zone of the spheroid, and (**b**) expression of CC chemokine receptor 4 (CCR4, green) in pancytokeratin (PCK, red)-positive cells located in the TME zone.

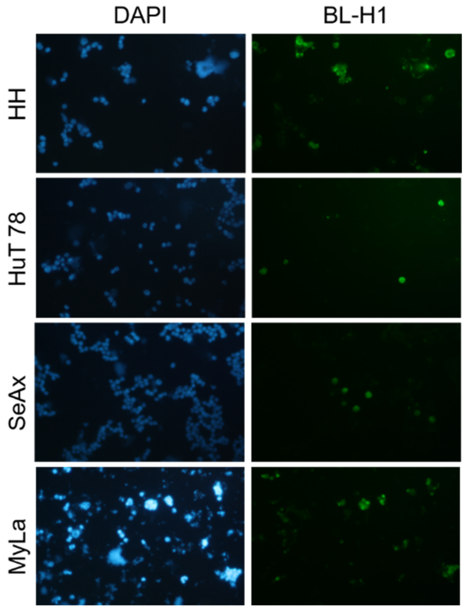

Supplementary Figure S5. Detection of H-1PV virions in cell lysates obtained from virus-treated heterotypic CTCL spheroids. Whole H-1PV virions were detected (BL-H1, green) on day three after infection in all CTCL spheroid lysates, in contrast to control monotypic non-CTCL (HUVEC) spheroids.

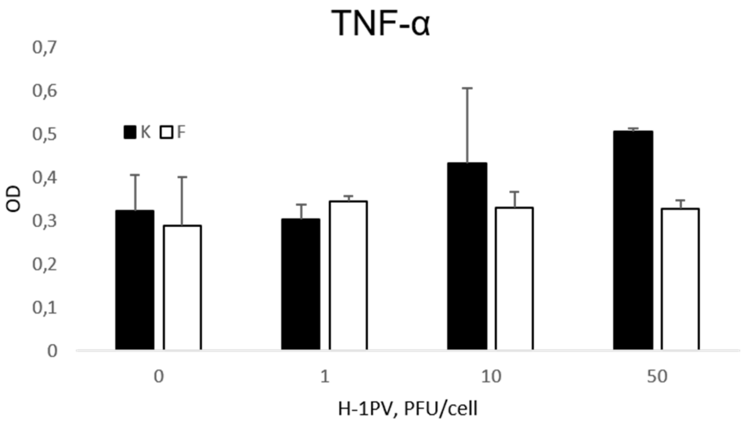

Supplementary Figure S6. Tumor necrosis factor-alpha release in co-cultures of keratinocyte-containing MyLa spheroids and healthy donor PBMCs. Increased TNF-α secretion was detected upon H-1PV infection of keratinocyte (K), but not fibroblast (F)-containing co-cultures. Further studies may be worth conducting, in order to substantiate this observation.

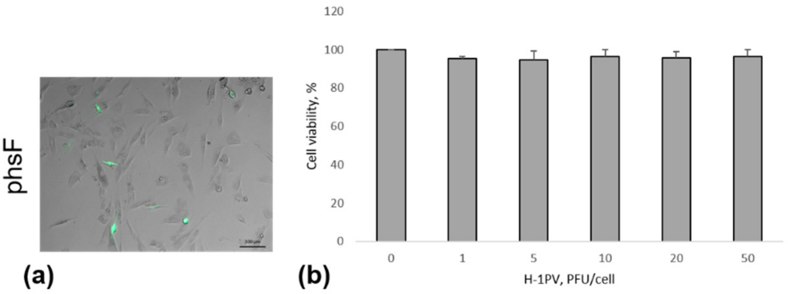

Supplementary Figure S7. H-1PV innocuousness for human primary healthy skin fibroblasts. Similar to various other normal controls (see main text), human healthy donor primary skin fibroblasts (phsF) (**a**) allowed H-1PV entry but (**b**) exhibited no sensitivity to virus-induced killing.

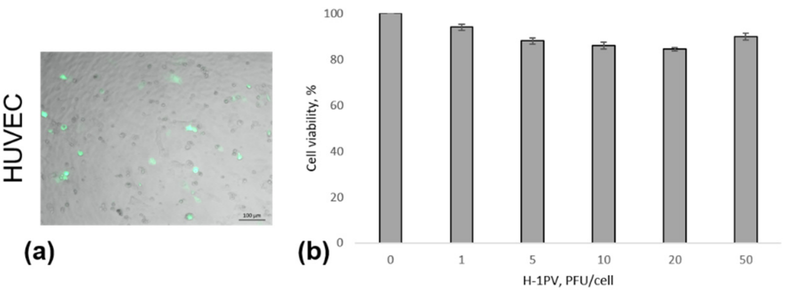

Supplementary Figure S8. H-1PV innocuousness for human umbilical vein endothelial (HUVEC) cells. Similar to the other normal controls (see main text), HUVEC cells (**a**) allowed H-1PV entry but (**b**) displayed insignificant responsiveness to virus-induced cytotoxicity.
